# With Bayesian estimation one can get all that Bayes factors offer, and more

**DOI:** 10.3758/s13423-022-02164-3

**Published:** 2022-09-09

**Authors:** Jorge N. Tendeiro, Henk A. L. Kiers

**Affiliations:** 1 Office of Research and Academia-Government-Community Collaboration, Education and Research Center for Artificial Intelligence and Data Innovation, Hiroshima University, Hiroshima, Japan; 2 University of Groningen, Groningen, Netherlands

**Keywords:** Bayes factor, Null hypothesis Bayesian testing, Bayesian estimation, Unification

## Abstract

In classical statistics, there is a close link between null hypothesis significance testing (NHST) and parameter estimation via confidence intervals. However, for the Bayesian counterpart, a link between null hypothesis Bayesian testing (NHBT) and Bayesian estimation via a posterior distribution is less straightforward, but does exist, and has recently been reiterated by Rouder, Haaf, and Vandekerckhove (2018). It hinges on a combination of a point mass probability and a probability density function as prior (denoted as the spike-and-slab prior). In the present paper, it is first carefully explained how the spike-and-slab prior is defined, and how results can be derived for which proofs were not given in Rouder, Haaf, and Vandekerckhove (2018). Next, it is shown that this spike-and-slab prior can be approximated by a pure probability density function with a rectangular peak around the center towering highly above the remainder of the density function. Finally, we will indicate how this ‘hill-and-chimney’ prior may in turn be approximated by fully continuous priors. In this way, it is shown that NHBT results can be approximated well by results from estimation using a strongly peaked prior, and it is noted that the estimation itself offers more than merely the posterior odds on which NHBT is based. Thus, it complies with the strong APA requirement of not just mentioning testing results but also offering effect size information. It also offers a transparent perspective on the NHBT approach employing a prior with a strong peak around the chosen point null hypothesis value.

In classical statistics, there is a close link between null hypothesis significance testing (NHST) and parameter estimation via confidence intervals. However, for the Bayesian counterpart, a link between null hypothesis Bayesian testing (NHBT) and Bayesian estimation via a posterior distribution is less straightforward, but does exist. This link between these two very important inferential strategies is actually of great relevance. The seemingly dichotomous divide between the adequacy of conducting a hypothesis test versus estimation often leads to polarized discussions and advice on how to choose between the two inferencial frameworks (e.g., Kruschke & Liddell, 2018b; Uygun Tunç, Tunç & Lakens, 2021; Wagenmakers et al., 2018).

Both Bayesian estimation and Bayesian hypothesis testing predicate on the uncontroversial Bayes’ theorem (e.g., Puga, Krzywinski, & Altman, 2015). On its own, the Bayes theorem offers a mathematical means of combining information from two distinct sources of information. *Prior distributions* may be used to encapsulate our current understanding of the phenomenon being studied, or to represent the predictions of the theory that we intend to test. The *likelihood function* captures the information provided by the data through the statistical model of choice. The combined information is summarized by the *posterior distributions*, which offer an updated account of our state of knowledge. We refer readers wishing to get started on basic Bayesian inference to a variety of introductory books and papers (e.g., Etz & Vandekerckhove, 2018; Gelman et al., 2013; Kruschke, 2015; van de Schoot et al., 2021).

Inference through Bayesian *estimation* consists of carefully describing the information shown in the posterior distribution, for instance, via *credible intervals* that attach probabilities to specific ranges of values for the parameter at hand. The Bayesian estimation approach is a widely recommended procedure for data analysis (e.g., Gelman et al., 2013; Kruschke, 2011; 2013; Kruschke & Liddell, 2018a, p. 170; Stern, 2016, p. 27; van der Linden & Chryst, 2017; van de Schoot et al., 2014). Bayesian hypothesis *testing*, on the other hand, compares the predictive ability of two competing models or hypotheses. A model that outperforms its competitor in predicting the observed data leads to a positively revised view of its ability to describe the phenomenon being studied. This update of the relative belief between the two models due to the observed data is processed, precisely, through the Bayes theorem.

The Bayesian testing approaches we refer to in this paper are based on comparing a point null hypothesis to an alternative hypothesis that specifies a parameter to be distributed according to a particular density function. We abbreviate this procedure as NHBT (null hypothesis Bayesian testing). The result is expressed in a so-called Bayes factor, which captures the evidence from the data for the point null hypothesis versus the specific alternative hypothesis chosen. This approach has been summarized by Kruschke and Liddell (2018a), who also issue five warnings as to its use (see also Tendeiro & Kiers, 2019, 2022; van Ravenzwaaij & Wagenmakers, 2021). A lot of attention nowadays is given to promoting and further developing Bayesian ways of testing null hypotheses. It is sometimes even suggested that testing should routinely precede estimation of effect sizes and their uncertainty in the inferential ladder, (e.g., Jeffreys 1961; van Ravenzwaaij & Wagenmakers, 2021; Wagenmakers et al., 2018).

It is not immediately clear how Bayesian estimation and testing, as described above, relate to each other. In fact, inferences derived from Bayesian estimation can easily lead to results that may appear to conflict with those from NHBT. For instance, when an NHBT is carried out by the programme (JASP Team, 2020), it also offers a posterior distribution and a highest density interval (HDI, also known as credible interval) based on that. An NHBT may indicate fairly strong evidence in favor of 0 while the HDI may not contain the value 0 (Kruschke & Liddell, 2018b; Tendeiro & Kiers, 2019). It is often argued that the researcher’s goals are what dictate the preference for either estimation or testing. This, however, has not lessened the discussion among campers on both ends of the spectrum.

One of the goals of this paper is to contribute towards a unification between estimation and testing in the Bayesian paradigm. We aim to do so in a manner such that it is accessible for the general reader who has only little knowledge of probability theory (but knows basic expressions). Such readers may skip the more technical parts, and notably the appendices; the latter clearly are for the experts. Furthermore, we are not the first ones to have attempted this unification. Of particular importance is the work of Rouder et al. (2018) and Liao, Midya, and Berg (2020). Rouder et al. (2018) drew attention to an earlier established link between Bayesian estimation and NHBT through the so-called spike-and-slab prior ‘density’ (Mitchell and Beauchamp, 1988). See also Kruschke (2018); a less detailed but related account on this is also given by Williams, Bååth, and Philipp (2017). Here, we explore the role of the spike-and-slab prior in Bayesian inference, and we reflect on how various *approximations* to the spike-and-slab prior allow a deeper understanding of the link between testing and estimation in Bayesian statistics. Such priors are by no means new. They feature strongly in the literature on selection of variables in regression. See O’Hara and Sillanpää (2009) for an excellent overview on this topic, and also George & McCulloch (1993; 1997), Ishwaran & Rao (2003; 2005), Kuo and Mallick (1998), Malsiner-Walli and Wagner (2011), Ntzoufras, Forster, and Dellaportas (2000), and Wagner and Duller (2012). Morey and Rouder (2011) mentioned such models in the simpler context of testing or estimating single parameters. Kruschke (2018, supplement) offers a more detailed analysis of such variants to the spike-and-slab prior. Thus, for the simple single parameter case, the literature has already surveyed various aspects of the concrete relation between estimation and Bayes factor based testing.

## Our contribution

This paper’s offering can be summarized in five main points. First, this paper further contributes towards an integrated view of estimation and testing, under the Bayesian paradigm. Second, we will identify the spike-and-slab prior as an extreme choice of a peaked prior, within a broad class of more or less peaked priors. With this purpose in mind, we will introduce what we dubbed the *hill-and-chimney* prior. Third, we will identify the Bayes factor and notably its related posterior odds, as only one of many interesting concrete measures to evaluate probabilities of events on the basis of a posterior distribution. Fourth, we will argue that the hill-and-chimney’s discontinuous nature is not ideal and then suggest continuous approximations. We discuss two relatively simple alternatives to this family of approximating continuous distributions. Fifth, we briefly discuss how our integrated inferential approach can be contextualized in the realm of decision theory, following Kruschke and Liddell (2018b). We end by summarizing our main ideas and discuss future avenues for research in the discussion.

All examples and figures can be reproduced by means of the accompanying R script available at the Open Science Framework (https://osf.io/6rt9m/). The repository also includes Stan and R scripts that readily allow fitting the estimation models advocated in this paper.

## Bayesian estimation

### Basics

**Fig. 1:**
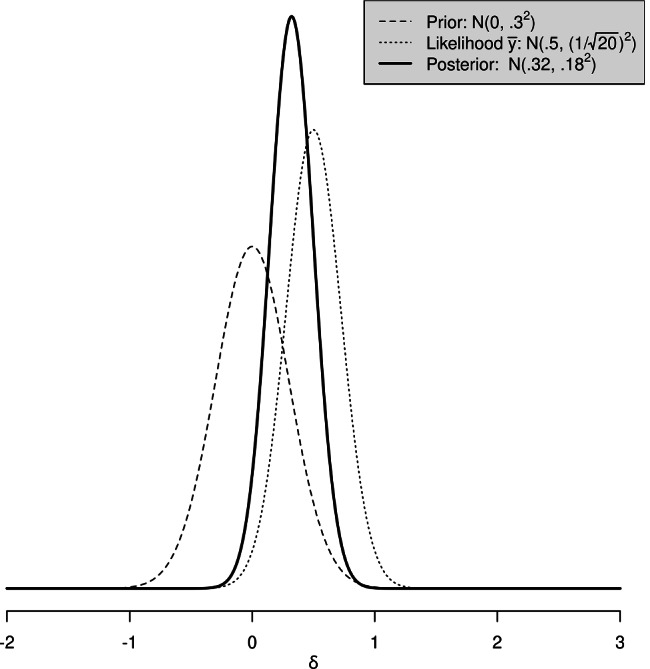
Bayesian estimation for parameter *δ*. Data are assumed to be normally distributed with (unknown) mean *δ* and standard deviation 1 in the population. The prior distribution is $\mathcal {N}(0, .3^{2})$. The sample of size 20 has sample mean equal to 0.5. The posterior distribution is the normalized product of the prior and the data distribution and it offers a compromise between both sources of information. We refer to Appendix A for the mathematical details of this posterior distribution

As in Rouder et al. (2018), consider data **y** = {*y*_1_,…,*y*_*n*_} of size *n*, assumed to be randomly sampled from a normal population with unknown mean *μ* and known variance *σ*^2^. Although quite unrealistic, the known variance assumption is used only because it facilitates the mathematical formulation and it does not bear consequences conceptually. Let *δ* denote a standardized effect size measure, defined as *μ*/*σ*, that is, the population mean divided by the population standard deviation. Our goal is to infer what can be said about the population effect size *δ*. For the Bayesian machinery to operate we must choose a prior for *δ*. Bayesian inference proceeds by combining the information in the prior with that from the data. The posterior distribution for *δ* will reflect our updated belief in *δ* in light of the observed data. As a simple example, consider Fig. 1. We assume that, for the population of interest, the variance is known and equal to 1. Our goal is to infer about the real value of *δ*. A prior normal distribution for *δ* with mean 0 and standard deviation .3 was chosen. Following Bayes’ theorem, this prior was multiplied by the normal likelihood based on a sample of size 20 and mean 0.5. The result—the posterior distribution—is the updated compromise for *δ* combining both the prior and the likelihood.

Of particular importance is the choice of the prior distribution. There are in fact many possible priors that we can choose from, depending on our purposes. What we propose to do in this paper—building a conceptual bridge between Bayesian estimation and testing—is based on using a special type of prior distribution that is not of common use within the social sciences: The so-called *spike-and-slab* prior.

### The spike-and-slab prior

**Fig. 2:**
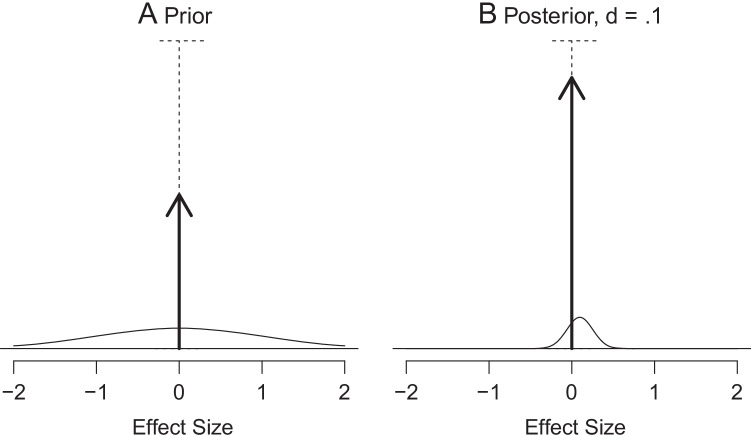
Visualization of the spike-and-slab model by Rouder et al. (2018), copied from their manuscript, with permission by the publisher. **A** Prior distribution on effect size *δ* = *μ*/*σ*, with half the mass in the spike and the slab centered around zero. **B** Posterior distribution on effect size *δ* for an observed effect size of *d* = .1 based on a sample size of 40

We consider the spike-and-slab prior following Rouder et al. (2018). For the idea behind this, they refer to Jeffreys (1939), while for the particular name of the model they refer to Mitchell and Beauchamp (1988). The name of this particular prior becomes obvious when looking at its plot: See Fig. 2A, which is a copy of Fig. 4A in Rouder et al. (2018). The spike-and-slab prior is a weighted combination of a probability density for all non-zero *δ* values and a probability mass at *δ* = 0 (in Fig. 2A, both parts have equal weight). The probability density is shown as the gentle symmetric curve in the bottom of the picture (which can be seen as a slab of sorts), while the arrow peaks above this curve (clearly a spike) and has half the length of the maximum indicated by the dashed horizontal bar. Note that the slab density and the spike probability are in different units, so the *y*-axis actually refers to two different scales. To understand what such a combination of a density and a probability mass function means, one could, for instance, assess that *P*(*δ* < 0) = .25, and likewise *P*(*δ* > 0) = .25. So we see that the three mutually exclusive events, *δ* < 0, *δ* = 0, and *δ* > 0, covering the whole possible range of outcomes, have probabilities .25, .50, and .25, respectively. Even though Fig. 2A does not give scale values for the density, for the above example, it is clear that the density should be such that the total area under the curve equals .5 (i.e., 1 minus the probability of the spike). For varying weights, the size of the height of the spike varies, and the height of the slab varies with it, ensuring that the total area under the curve always equals one minus the probability mass at 0.

Mathematically, the spike-and-slab prior can be given as follows:^1^

1 $$\left\{\begin{array}{lll} p(\delta) & = (1-\rho_{0})\times\frac{1}{\sqrt{2\pi}\sigma_{0}}\exp\left(-\frac{\delta^{2}}{2{\sigma_{0}^{2}}}\right) & \text{, if } \delta\not=0 \\ P(\delta=0) & = \rho_{0} & \text{, if } \delta=0 \end{array}\right..$$

Please note that *p*(*δ*) denotes a (non-normalized) density function which is proportional to the $\mathcal {N}(0,{\sigma _{0}^{2}})$ distribution. The variance ${\sigma _{0}^{2}}$ controls the spread of the slab part of the prior. *P*(*δ* = 0), on the other hand, is a probability, hence the density function values are incomparable to the values of the probability.^2^

Considering the above as the specification of the prior for all values of *δ*, the next step in estimation is to observe data and establish the posterior distribution for *δ* given these data. Then we have to work separately for *δ*≠ 0 and *δ* = 0. For *δ*≠ 0, we compute the product of the likelihood and the prior density functions and denote this as the nonnormalized posterior density distribution for *δ*≠ 0. For *δ* = 0, we compute the product of *ρ*_0_ and the likelihood at *δ* = 0 and consider this the nonnormalized posterior probability for *δ* = 0. Finally, we obtain the full posterior distribution by normalizing the combination of the nonnormalized parts mentioned above. Rouder et al. (2018) say “It is straightforward to update beliefs about *δ* in the spike-and-slab model using Bayes’ rule”, and then give the posterior in a footnote. For our own interest, but possibly also the reader’s interest, we completely derive this result in Appendix C (and while doing so made a correction).

### The hill-and-chimney prior

In general, when estimating a posterior density, the researcher is asked to specify a prior and justify its choice. A common justification is that the prior gives a good representation of the current knowledge and/or the researcher’s belief on the probability density of the parameter of interest. An alternative justification is that a prior should be objective, that is, it should not incorporate a researcher’s subjective knowledge or beliefs. The idea is that priors are chosen without committing with too specific information on the parameters of interest (for instance, a wide normal distribution, which ensures that impossible extreme values get low prior densities, while the whole range of realistic values get very similar prior densities). In this way, objective priors are still subjective, or maybe ‘intersubjective’ (i.e., tentatively agreed upon by a number of people) choices of appropriate almost ‘uninformative’ priors.

Now as to the spike-and-slab prior, if indeed a researcher has reasons to believe that the value of exactly 0 has a much higher chance of being true than any other values (even values extremely close to 0), the spike-and-slab model offers an appropriate type of prior. It treats the value 0 as qualitatively different from other values for the parameter. We, however, doubt whether the value 0 will often, if ever, have such a special status (see Cohen, 1994; Meehl, 1978; Vardeman, 1987). If choosing such a prior, we think that the researcher must come up with a good reasoning why this could represent his/her current belief and knowledge on the probability distribution of the parameter of interest. This could be difficult without recourse to hard theoretical reasonings, and the latter, unfortunately, do not seem available in behavioral sciences (e.g., Oberauer and Lewandowsky, 2019). The examples often encountered when defending the point null hypothesis are in the realm of extrasensory perception, or precognition (e.g., see Wagenmakers et al., 2018), where on purely theoretical grounds it is considered that exact 0 effects should have a high probability,^3^ but to us such situations seem rare in actual behavioral science research.

**Fig. 3:**
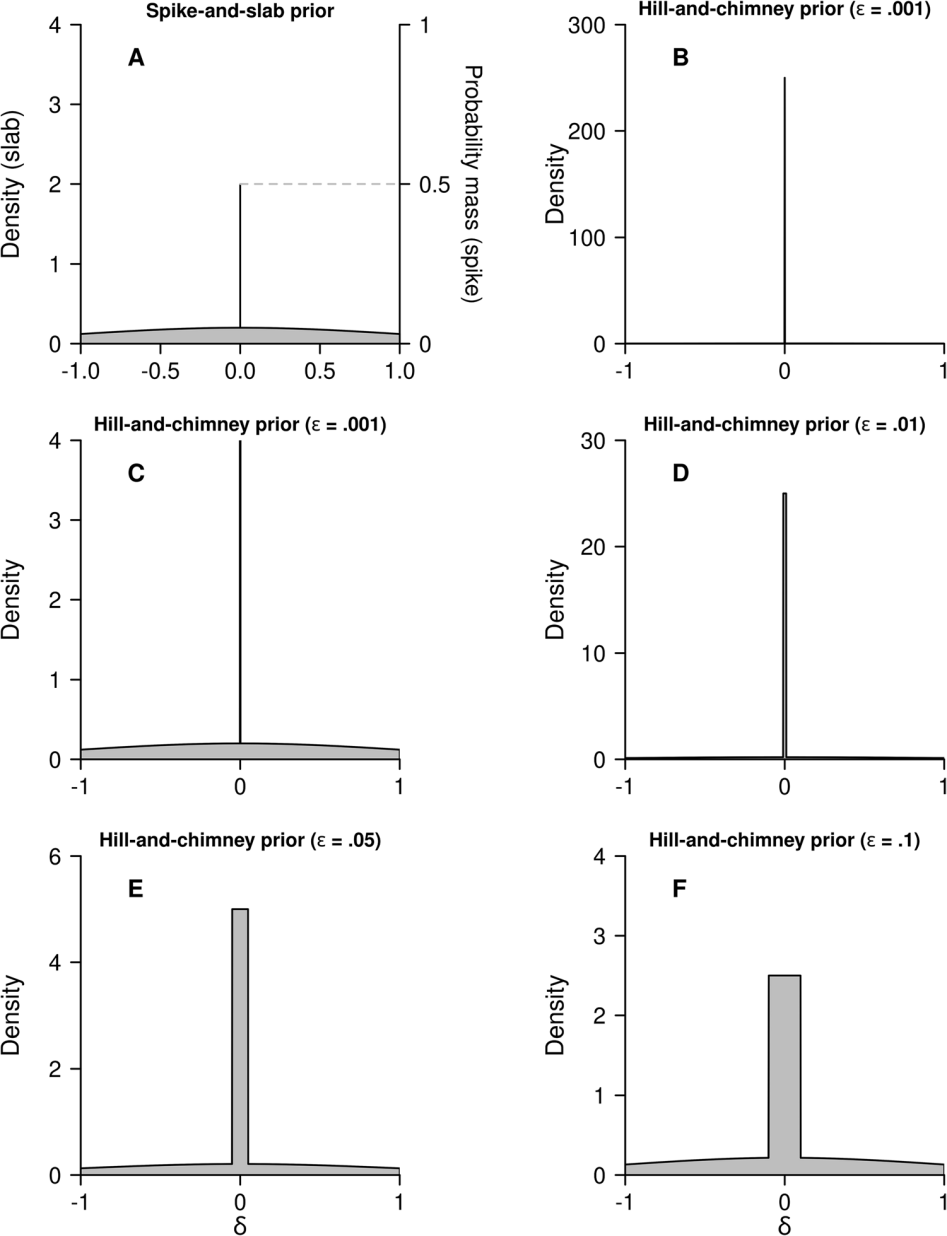
The spike-and-slab prior (**A**), and the chimney-and-hill prior for *ε* = .001 (**B**); panel **C** displays the same prior as panel **B**, but with the scale of the *y*-axis set equal to that of panel **A**, so note that the ‘chimney’ actually continues with more than 60 times the height displayed; the next panels show chimney-and-hill priors for *ε* = .01 (**D**), *ε* = .05 (**E**), and *ε* = .1 (**F**). All priors were based on *ρ*_0_ = .5 and *σ*_0_ = 1. The spike-and-slab prior is based on multiplying the $\mathcal {N}(0,1)$ density by .5, and the spike is supposed to represent .5 probability on *δ* = 0. In all cases, the priors have been displayed on the ranges [− 1,1]. Note that the scales of the density on the *y*-axes differ markedly

In practice, rather than believing with a sizeable probability that there is exactly no effect at all (even in a population), it seems to make more sense to believe with sizeable probability that the effect is merely *negligible* for all practical purposes (e.g., Blume, Greevy, Welty, Smith, & Dupont, 2019). Depending on these practical purposes this could mean, for instance, a mean effect size in the interval [−.00001,.00001] or in the interval [−.01,.01]. In fact, 0 is often used as a simplified proxy for such negligible effects. A practically realistic approximation to the spike-and-slab prior could be what we here call the *hill-and-chimney* prior (see Fig. 3); the term “chimney” was chosen to replace the word spike, as the spike refers to a line or if you like an extremely narrow rectangle, while the chimney has a noticeable, but still practically negligible width; the term “hill” to replace slab was chosen to come up with a landscape-like metaphor. Rather than assigning probability mass to a spike, the mass is now assigned to an interval, and the graph can now actually express a density across the whole width of this interval. Let the interval of negligibility generally be defined as [−*ε*,*ε*] where *ε* = .00001 or *ε* = .01 in the above two examples. Then the hill-and-chimney prior can be defined as

2 $$p(\delta) = \left\{\begin{array}{ll} k\times\frac{1}{\sqrt{2\pi}\sigma_{0}}\exp\left(-\frac{\delta^{2}}{2{\sigma_{0}^{2}}}\right) & \text{, if } \delta\not\in[-\varepsilon,\varepsilon] \\ \frac{\rho_{0}}{2\varepsilon} & \text{, if } \delta\in[-\varepsilon,\varepsilon] \end{array}, \right.$$

where the value *k* normalizes the function such that the sum of the integrals over $[-\infty , -\varepsilon ]$ and $[\varepsilon , \infty ]$ equals (1 − *ρ*_0_), and hence, using that the integral over [−*ε*,*ε*] equals *ρ*_0_, the integral of the function over $[-\infty ,\infty ]$ equals 1, which shows that it gives a proper density function (see Appendix D).

To make this more concrete, let’s take a look at the six panels of Fig. 3, displaying priors based on *ρ*_0_ = .5 and *σ*_0_ = 1. First of all, panel Fig. 3A shows the spike-and-slab prior. In contrast to Fig. 2A by Rouder et al. (2018), we distinguish the two sets of units at stake here: Those for the slab and those for the spike, and indicate these alongside the *y*-axes. We have here chosen to take a length of the spike roughly comparable to that in Fig. 2A.

Panels Fig. 3B-F show the hill-and-chimney prior density functions for various values of *ε*. One might expect that the one for the smallest value of *ε* (i.e., *ε* = .001) is the most similar to the spike-and-slab prior. The prior in panel Fig. 3B, however, looks quite different compared to the spike-and-slab prior, as the curvy base area seems missing. This is due to the fact that the chimney has been displayed in its full height, which required shrinking the *y*-axis considerably. Upon zooming in (panel Fig. 3C), now clearly the slightly curvy surface is visible and actually very similar to that in panel Fig. 3A. Because the hill-and-chimney approximation employs the same units for the hill and the chimney, we see that the density in the narrow area around 0 is many times higher than the density just outside it. For instance, the density for *δ* = .001 is roughly 1000 times as high as the density for the closely neighboring value *δ* = .0011 or the even closer *δ* = .001000001. The spike-and-slab visualization in Figs. 2A and 3A does not reveal this, because the display represents two superimposed incomparable entities in a single graph. By approximating the spike-and-slab prior by the hill-and-chimney prior, we can correctly interpret the plot of superimposed graphs, and we see that for narrow chimneys their heights are immense. In the next section, it will be demonstrated that indeed the hill-and-chimney prior in the limit as *ε* approaches 0, equals the spike-and-slab prior.

For now, it is interesting to also inspect the other panels of Fig. 3. Panels Fig. 3D, E, and F display hill-and-chimney priors for increasing chimney widths, that is, for *ε* = .01, *ε* = .05, and *ε* = .1, respectively. It can be seen that for *ε* = .01, the chimney still strongly dominates the hill, while for *ε* = .1 this is no longer strongly the case. However, the interval [−.1,.1] can hardly be considered similar to a spike at 0 (Fig. 3F). It could, however, represent a proper way of representing a somewhat strong belief in values of the effect size that are of little or no practical value. One last remark on the hill-and-chimney priors: The ones displayed here have been based on equal probabilities for the chimney and the joint area to the right and left of it. That is, in the plots we took *ρ*_0_ = .5. Of course, the degree of dominance of the chimney over the hill would diminish if smaller values for *ρ*_0_ would be taken, and increase if larger values would be taken. If, for instance, one would take *ρ*_0_ = .001, the chimney in panel Fig. 3C would roughly be at the same height as the curve, and hence no longer dominate it.

**Fig. 4:**
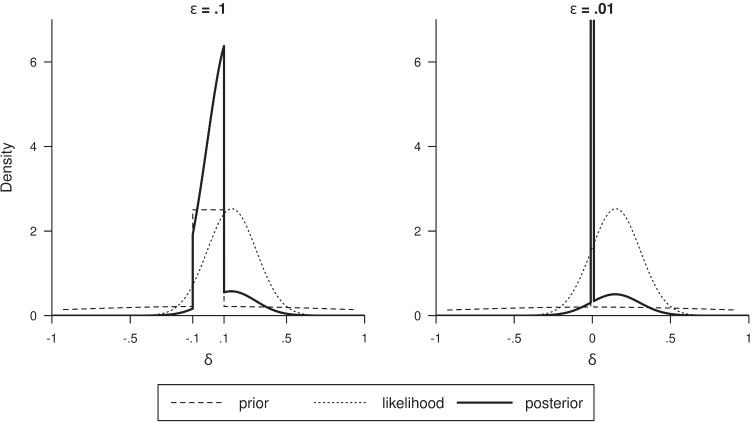
Two hill-and-chimney priors (*dashed curves*), with associated posteriors (*solid curves*) and likelihood (*dotted curve*), for data from a sample of size 40 and with mean effect size .15. The left panel is based on a chimney for *ε* = .1 (i.e., width = .2) and the right panel is based on a chimney for *ε* = .01 (i.e., width = .02)

Next, it is interesting to see what the full posterior distribution looks like using these hill-and-chimney priors. A derivation of this is given in Appendix D. Two examples are displayed in Fig. 4. Both examples are based on a sample of size 40 and mean of **y** equal to .15, but the difference is in the choice of *ε*. In the left panel *ε* = .1, and we see a fairly broad chimney for the prior (dashed curve), while in the right panel *ε* = .01, yielding a much narrower chimney. The posterior for the broad chimney has a somewhat jagged shape, and peaks fairly strongly above − .1 and .1, but it can also be seen that there is a nonnegligible posterior probability mass to the right of .1, which equals *P*(*δ* > .1|**y**) = .14. Given the posterior distribution, probabilities for any other range of values of *δ* can be computed: For instance, *P*(*δ* > 0|**y**) = .68, showing that the probability that *δ* is positive clearly exceeds that of *δ* being negative. If for some practical reason only effect sizes above .3 are to be taken seriously, it is worthwhile to know that *P*(*δ* > .3|**y**) = .04, hence it is quite improbable that the effect size is actually relevant in light of this practical relevance threshold. Also, the probability of *δ* lying in a small interval is interesting. Such an interval could be a “region of practical equivalence” (ROPE) around the value 0 (e.g., see Kruschke, 2018), which could be the interval [−.3,.3]. We can directly compute *P*(*δ* ∈ [−.3,.3]|**y**) = .96, meaning that, if values within the interval [−.3,.3] refer to negligible (downward or upward) effects, then the probability that the effect is negligible either way is as high as .96. However, possibly the ROPE should be taken much narrower, for instance, as narrow as the chimney. In that case, we compute *P*(*δ* ∈ [−.1,.1]|**y**) = .85, which shows that the probability of *δ* being within the area defined by the chimney is still quite high.

## Null hypothesis Bayesian testing (NHBT)

By null hypothesis Bayesian testing we denote a particular method that allows comparing two hypotheses or models: A point null hypothesis and an alternative hypothesis specifying a range of parameter values, for which a within-hypothesis prior density distribution has been specified. Below, we start by introducing the Bayes factor, and then consider how the Bayes factor can be interpreted directly in terms of the posterior distribution when the spike-and-slab prior has been employed. The spike-and-slab prior will finally allow us to draw a full circle connecting Bayesian estimation and NHBT.

### The Bayes factor

The Bayes factor for ${\mathscr{H}}_{1}$ versus ${\mathscr{H}}_{0}$, defined by $B_{10}=p(\mathbf {y}|{\mathscr{H}}_{1}) / p(\mathbf {y}|{\mathscr{H}}_{0})$, is a measure of the relative predictive ability of both models. The Bayes factor *B*_10_ is interpreted as a measure of evidence for ${\mathscr{H}}_{1}$ versus ${\mathscr{H}}_{0}$ provided by the data, and the evidence is in favor of ${\mathscr{H}}_{1}$ when *B*_10_ > 1, and for instance, strongly in favor of ${\mathscr{H}}_{1}$ if *B*_10_ > 10 (Jeffreys, 1961). The definition of *B*_10_ implies that $B_{01}=p(\mathbf {y}|{\mathscr{H}}_{0}) / p(\mathbf {y}|{\mathscr{H}}_{1}) = 1/B_{10}$.

**Fig. 5:**
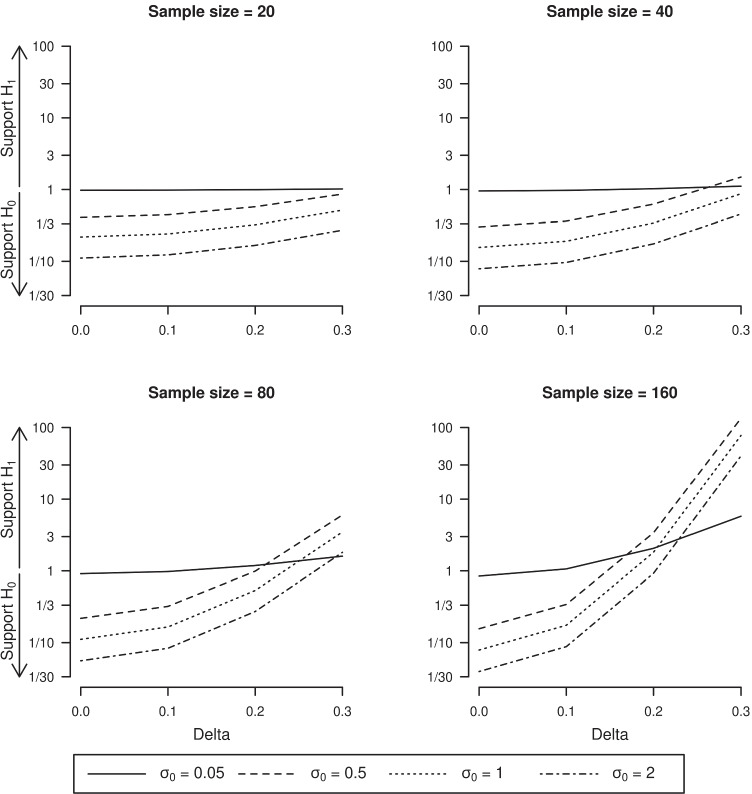
The one-sample *t* test Bayes factor computed as in JASP (${\mathscr{H}}_{0}:\delta =0$ versus ${\mathscr{H}}_{1}: \delta \sim \mathcal {N}(0, {\sigma _{0}^{2}})$), for various sample sizes (20, 40, 80, 160), observed effect sizes (*d* = 0, .1, .2, .3), and priors ($\mathcal {N}(0, {\sigma _{0}^{2}})$ for *σ*_0_ = 0.05, 0.5, 1, 2). Data were randomly sampled from a normal distribution such that their sample mean and standard deviation are equal to *d* and 1, respectively. The *y*-axis is on the log-scale

There are now various software packages that allow computing Bayes factors for a wide range of methods (e.g., the R bayesfactor package; Morey and Rouder, 2018; JASP Team, 2020). JASP in particular offers a very intuitive GUI that facilitates computing Bayes factors for a wide range of informative priors. For illustration, we give the results by JASP for a series of one-sample *t* tests for various sample sizes, effect sizes, and priors; see Fig. 5.^4^ For the standardized effect size *δ* we used the $\mathcal {N}(0, {\sigma _{0}^{2}})$ prior available in JASP, for several values of the standard deviation *σ*_0_. Other common options for the prior available in JASP are the Cauchy and the *t*-distribution. As can be seen from Fig. 5, the larger the effect on the *x*-axis, the more the evidence in favor of ${\mathscr{H}}_{1}$, and this relationship strengthens with sample size. Also, broad priors under ${\mathscr{H}}_{1}$ (i.e., larger *σ*_0_ values) are associated with stronger evidence in favor of the null hypothesis. This happens because broad priors dilute the predictive ability of model ${\mathscr{H}}_{1}$ over a wide range of parameter values, thus hurting the model’s ability to predict the observed data. It is therefore important to choose priors under ${\mathscr{H}}_{1}$ judiciously.

The Bayes factor is seen as “strength of evidence from the data about the models, precisely because the strength of evidence should refer to how data lead to revision of beliefs.” (see Rouder et al., 2018, p. 105, referring back to Morey, Romeijn, & Rouder, 2016 and Jeffreys, 1961). Indeed, the Bayes factor is the factor transforming given prior odds $P({\mathscr{H}}_{1})/P({\mathscr{H}}_{0})$ into posterior odds $P({\mathscr{H}}_{1}|\mathbf {y})/P({\mathscr{H}}_{0}|\mathbf {y})$, where here the probabilities refer to probabilities that the hypotheses ${\mathscr{H}}_{1}$ and ${\mathscr{H}}_{0}$, respectively, are true. This is because $P({\mathscr{H}}_{1}|\mathbf {y})/P({\mathscr{H}}_{0}|\textbf {y})= B_{10}\times P({\mathscr{H}}_{1})/P({\mathscr{H}}_{0})$, and indeed the role of the Bayes factor is bringing in information from the observed data. So the Bayes factor can be seen as an abstract and general measure of evidence from the data, but it does not lead by itself to concrete conclusions on how probable it is that either ${\mathscr{H}}_{1}$ or ${\mathscr{H}}_{0}$ is true. For such conclusion drawing and/or decision-making one will then need to specify the prior odds^5^ and compute the posterior odds. If, for example, the prior odds are set to 10/90, and *B*_10_ = 10, then the posterior odds are 100/90, so only slightly more in favor of ${\mathscr{H}}_{1}$ than of ${\mathscr{H}}_{0}$, and clearly not by the 10/1 ratio the Bayes factor might seem to suggest. Only if the prior odds are set to 50/50, the posterior odds equals the Bayes factor and only then the Bayes factor has an appealing interpretation for conclusion drawing. Later in this paper, we will also use equal prior odds, but at the same time we want to stress that 50/50 is not any more reasonable than any other prior odds (cf. Hinkley, 1987; Kruschke & Liddell, 2018a).

The NHBT procedure may seem to be a direct alternative for NHST, which compares ${\mathscr{H}}_{0}:\delta =0$ versus ${\mathscr{H}}_{1}:\delta \not =0$, but, as Wagenmakers et al. (2018) put it “In Bayesian statistics, this alternative hypothesis needs to be specified exactly.” This means that the hypothesis does not just state that *δ*≠ 0, but it gives an exact density distribution for *δ* under ${\mathscr{H}}_{1}$. This should be reflected in the process of drawing conclusions, which should not be phrased in terms of evidence for *δ* = 0 or *δ*≠ 0, but in terms of “higher (or lower) probability that *δ* = 0 than that *δ* has a normal distribution with mean 0 and variance ${\sigma _{0}^{2}}$.” In actual practice, where for instance one is testing the effect of a psychological treatment, formulating the results of a study as “it was found that the probability that the population treatment effect is normally distributed with mean 0 and variance 2 is 12.1 times higher than that the effect of the treatment is 0”, however, will not appeal to the reader, and one will easily resort to statements like “it was found that the probability that there is a treatment effect is 12.1 times higher than that there is not.” The conclusion is that it seems indeed difficult to deal with the notions of posterior and prior probability of ${\mathscr{H}}_{1}$, given that ${\mathscr{H}}_{1}$ itself is probabilistic in nature. Luckily, the spike-and-slab prior helps to conceptually bring these concepts together.

### The spike-and-slab prior in NHBT

We can conceive of using the spike-and-slab prior given by Eq. 1 under the alternative hypothesis ${\mathscr{H}}_{1}$. This allows combining the two types of probabilities that we alluded to above. The spike-and-slab prior can be regarded as a weighted sum such that the weights are the prior model probabilities: $\rho _{0}=P({\mathscr{H}}_{0})$ and hence $1-\rho _{0}=P({\mathscr{H}}_{1})$ (similarly for the spike-and-slab posterior, with $\rho _{1}=P({\mathscr{H}}_{0}|\mathbf {y})$ and $1-\rho _{1}=P({\mathscr{H}}_{1}|\mathbf {y})$; see Appendix C). The spike-and-slab prior effectively renders a Bayes model averaged posterior (Hoeting, Madigan, Raftery, & Volinksy, 1999), since the weights of the spike and the slab posterior components correspond to the posterior probabilities of either model. A specification of the probability (density) for each value of *δ* can be obtained by multiplying the probabilities at stake. That is, for *δ* = 0 the probability simply is *P*(*δ* = 0) = *ρ*_0_, because its probability is specified fully by $P({\mathscr{H}}_{0})$, while to obtain *p*(*δ*) for other values of *δ*, we multiply the density specified *within* ${\mathscr{H}}_{1}$, that is $p(\delta |{\mathscr{H}}_{1})$, by the probability *of* ${\mathscr{H}}_{1}$ to be true (P$({\mathscr{H}}_{1})$). Starting from this prior specification, and then estimating the associated posterior probability distribution, leads to a very insightful interpretation of NHBT. Rouder et al. (2018) recently reviewed this earlier noticed intricate relation between NHBT and parameter estimation. In the next section, we will discuss this relation and prove an interesting approximate relation to priors that are easier to handle.

In line with our running example, let us consider ${\mathscr{H}}_{0}:\delta =0$ versus ${\mathscr{H}}_{1}:\delta \sim \mathcal {N}(0,{\sigma _{0}^{2}})$ for known *σ*. Rouder et al. (2018) give, without proof (but see our Appendix B for a proof), an expression for *B*_10_ for ${\mathscr{H}}_{0}$ and ${\mathscr{H}}_{1}$ defined above in terms of $d=\overline {y}/\sigma$, *n*, and *σ*_0_.

## Relation between NHBT and the spike-and-slab prior

Rouder et al. (2018, p. 108) point out that “there is an intimate relationship between the spike-and-slab posterior distribution and the Bayes factor.” Indeed, upon observing that

3 $$\underset{\text{posterior odds}}{\underbrace{\frac{P(\mathcal{H}_{1}|\mathbf{y})}{P(\mathcal{H}_{0}|\mathbf{y})}}} = B_{10} \underset{\text{prior odds}}{\underbrace{\frac{P(\mathcal{H}_{1})}{P(\mathcal{H}_{0})}}}$$

we have that

4 $$\frac{1-\rho_{1}}{\rho_{1}} = B_{10} \frac{1-\rho_{0}}{\rho_{0}},$$

which neatly demonstrates how the Bayes factor relates to the spike-and-slab prior and posterior distributions. However, the spike-and-slab posterior has much more to offer than the posterior odds above: It is possible to compute posterior probabilities for all sorts of ranges of values of *δ*. So the spike-and-slab based estimation procedure implicitly associated with NHBT actually leads to more information than what NHBT gives on its own. This can be gleaned from Fig. 2B, which visualizes the full posterior for a particular data set (in this case, with $\overline {y}=.1$ and *n* = 40, assuming *σ* = 1). Here we see that the spike has grown somewhat, while the slab has become less wide and has a smaller surface area under it. From the figure we could conclude, for instance, that *P*(*δ* > .5|**y**) ≈ 0, in other words, the probability that the effect size is larger than .5 is negligible. Also, we can assess that *P*(*δ* > 0|**y**) = .12, while *P*(*δ* < 0|**y**) = .04. From NHBT alone, no such conclusion could be drawn. Thus, estimation of the posterior distribution based on the spike-and-slab prior offers the same information as NHBT does, and more.

This relation between NHBT and the spike-and-slab model is bidirectional. We have already mentioned that the spike-and-slab model is a weighted mixture of both hypotheses being tested. Conversely, given the spike-and-slab model, it is simple to derive the hypotheses being tested (Liao et al., 2020, pp. 2–3). However, this is only possible when we know ahead of time the parameter values associated to each hypothesis. The spike-and-slab mixed model could otherwise be arrived at in many different ways, depending on which of its components were being weighed in together (Kruschke, 2018). Knowing the parameter supports across hypotheses prevents such ambiguity from arising.

We have now seen that posterior density estimation with the spike-and-slab prior offers all information needed for NHBT. That is, using the spike-and-slab prior, from the resulting posterior density one can directly assess *B*_10_ as

$$\frac{P(\delta\not=0|\mathbf{y}) / P(\delta=0|\mathbf{y})}{(1-\rho_{0})/\rho_{0}},$$

which in the special case of prior odds equaling 1, reduces to *B*_10_ = *P*(*δ*≠ 0|**y**)/*P*(*δ* = 0|**y**). Next, we show how the spike-and-slab and hill-and-chimney priors are related and how that is of interest for the Bayes factor.

## Relation between spike-and-slab and hill-and-chimney priors and posteriors

In the present section, we demonstrate to what extent using spike-and-slab priors comes down to the same as using hill-and-chimney priors. We do so by offering a formal proof (see Appendix D) to the following intuitive result: *The hill-and-chimney prior converges to the spike-and-slab prior as* *ε* *converges to 0.* As a result, a limiting relation between the posterior distributions resulting from the two different priors must also necessarily hold.

It then follows that the posterior odds based on the hill-and-chimney prior, $\frac {P(\delta \not \in [-\varepsilon ,\varepsilon ]|\mathbf {y})}{P(\delta \in [-\varepsilon ,\varepsilon ]|\mathbf {y})}$, will, in the limit of $\varepsilon \rightarrow 0$, equal the posterior odds based on the spike-and-slab prior (see Eq. 4). In other words,

5 $$\underset{\text{spike-and-slab}}{\underbrace{B_{10} \frac{1-\rho_{0}}{\rho_{0}} = \frac{P(\delta\not=0|\mathbf{y})}{P(\delta=0|\mathbf{y})}}} \simeq \underset{\text{hill-and-chimney}}{\underbrace{\frac{P(\delta\not\in[-\varepsilon,\varepsilon]|\mathbf{y})}{P(\delta\in[-\varepsilon,\varepsilon]|\mathbf{y})}}},$$

where the approximation improves as *ε* approaches 0.

**Fig. 6:**
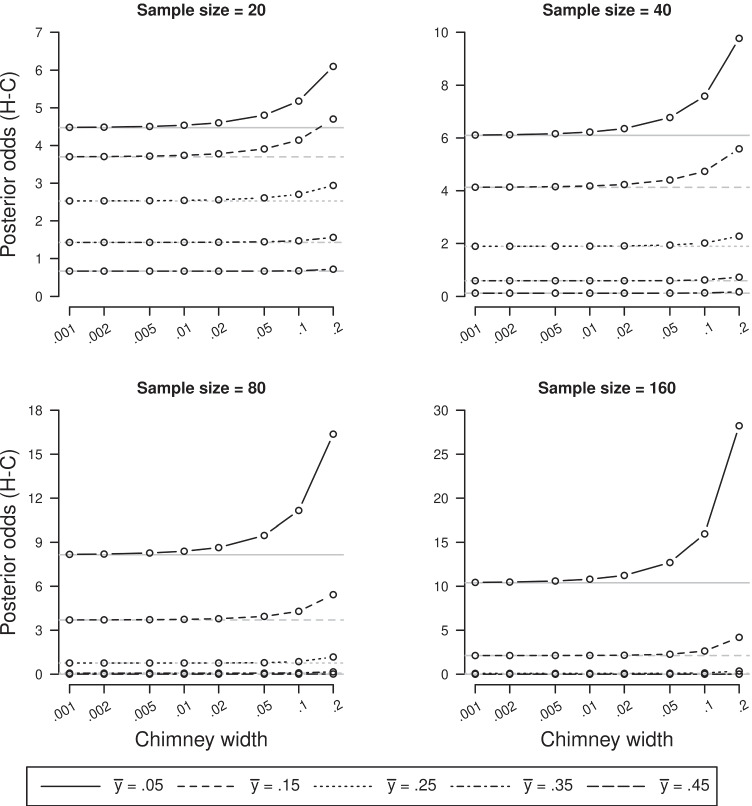
Posterior odds (*vertical axis*) obtained using hill-and-chimney priors plotted against the various chimney widths (*horizontal axis*, log-scale), for varying sample sizes (20, 40, 80, 160) and mean values of *y* (.05, .15, .25, .35, .45). *Grey horizontal lines* represent the values of the associated spike-and-slab posterior odds

For practical purposes, it is interesting to get some feeling for how quickly this happens. As an example, we consider data for which $\overline {y} =$.05, .15, .25, .35, and .45, and we assume *σ* = *σ*_0_ = 1 and prior odds equal to 1. The chimney width is gradually decreased from 0.2 to 0.001, and results were compared with the posterior odds for the spike-and-slab model, which now equal the Bayes factor. To simplify plotting, we focus on the posterior odds $\frac {P(\delta \in [-\varepsilon ,\varepsilon ]|\mathbf {y})}{P(\delta \not \in [-\varepsilon ,\varepsilon ]|\mathbf {y})}$ and thus on *B*_01_, rather than *B*_10_. Figure 6 displays the results of these analyses for four different sample sizes, where the posterior odds is displayed on the *y*-axis against the various chimney widths. It can be seen that indeed, as the chimney width decreases, the posterior odds approaches *B*_01_. The speed of the approximation depends on the sample size and on the value of $\overline {y}$, but from all figures it can be seen that substantial differences are seen for the chimney widths from .02 on (albeit only for small effects sizes), while up to .01 differences could be considered negligible.

As mentioned, the hill-and-chimney prior does not approximate the spike-and-slab prior very closely for *ε* = .1. For example, we can again consider the example previously discussed through Fig. 4. The posterior odds *P*(*δ* ∈ [−*ε*,*ε*]|**y**)/*P*(*δ*∉[−*ε*,*ε*]|**y**) equals 5.59 for *ε* = .1. This is an approximation to the Bayes factor (associated with the spike-and-slab prior) assuming prior odds equal to 1. A better approximation is obtained for *ε* = .01, as is displayed in the right-hand panel of Fig. 4. Now the posterior odds is 4.23, which is indeed very close to the Bayes factor *B*_01_ = 4.13. The hill-and chimney plot of the prior (dashed line) can be seen to display a far protruding spike (which actually extends till 25), and the posterior is even more spiky, with a top at 42.9. Also here, it can be seen that there still is some mass for values higher than *ε* = .01 (i.e., to the right of the spike): *P*(*δ* > .01|**y**) = .16. Furthermore, one could verify the probability within the ROPE interval [−.3,.3], which now is *P*(*δ* ∈ [−.3,.3]|**y**) = .97 or within the interval [−.1,.1], which now is *P*(*δ* ∈ [−.1,.1]|**y**) = .87. Interestingly, what we learn now is that, although the two panels in Fig. 4 look at first sight quite different, probability statements for even quite small intervals give almost the same values (recall that, based on Fig. 4A, *P*(*δ* ∈ [−.3,.3]|**y**) = .96 and *P*(*δ* ∈ [−.1,.1]|**y**) = .85). Furthermore, we see that it is easy and worthwhile to inspect more information from the posterior distribution than just the posterior odds. When researchers indeed wish to specify a prior in a spike-like way, using the approximating hill-and-chimney prior could be a good choice which displays all aspects of the prior in a comparable way, that is, in terms of densities. The hill-and-chimney prior, however, still seems unrealistic because of its discontinuity (around the chimney), which also leads to a strangely jagged and discontinuous posterior distribution. In the next section, it will be shown that smooth prior density functions can be found that approximate these hill-and-chimney priors closely.

## Approximating hill-and-chimney priors by smooth prior distributions

As mentioned earlier, the hill-and-chimney priors still have a discontinuity at the interval boundaries. It seems desirable to replace it by a continuous density function, so as to avoid that, for instance, prior densities at *δ* = .001 and *δ* = .0011 differ enormously.^6^ Frühwirth-Schnatter and Wagner (2011) and Wagner and Duller (2012) also discussed a similar search for absolutely continuous distributions in the realm of Bayesian variable selection. Liao et al. (2020) further argued that, in some cases, discontinuous *ε*-boundary points are associated with poor performance of the Bayes factor, in contrast with the posterior odds. An efficient way of approximating the hill-and-chimney prior distribution (Eq. 2) by a smooth continuous density is based on *mollification* (Friedrichs, 1944). The idea consists of considering a so-called mollifier function. The mollifier allows smoothing an irregular or even non-differentiable function by an infinitely differentiable function. The approximation works to a high degree of accuracy.

We first defined the mollifier function as follows^7^:

6 $$\varphi_{\alpha}(\delta) = \left\{ \begin{array}{ll} k\exp\left[\left(\frac{\alpha}{\varepsilon_{\sigma_{0}}}\right)^{2} + \frac{1}{(\delta/\alpha)^{2}-(\varepsilon_{\sigma_{0}}/\alpha)^{2}}\right], & |\delta| < \varepsilon_{\sigma_{0}} \\ 0, & |\delta| \geq \varepsilon_{\sigma_{0}} \end{array} \right..$$

The scale parameter *α* is a positive quantity that tunes the degree of smoothing attained: The larger, the closer the approximation (but too large values may also lead to numerical instability). The value $\varepsilon _{\sigma _{0}}$ should be some value at least larger than *ε* (the upper limit of the chimney), in order to allow the approximation around the chimney to work well (in our computations we used $\varepsilon _{\sigma _{0}}=4\sigma _{0}$). Some trial and error to find optimal values for *α* and $\varepsilon _{\sigma _{0}}$ is usually required. Constant *k* adjusts the function so that $\displaystyle {\int \limits }_{\mathbb {R}}\varphi _{\alpha }(\delta ) d\delta = 1$.

Next, using this mollifier, a continuous, smoothed, hill-and-chimney prior, denoted below as *f*_*α*_(*δ*), is defined as the convolution between the hill-and-chimney prior *p*(*δ*) (Eq. 2) and the mollifier in Eq. 6:

7 $$f_{\alpha}(\delta) = {\int}_{\mathbb{R}} p(t)\varphi_{\alpha}(\delta-t)\; dt\;.$$

**Fig. 7:**
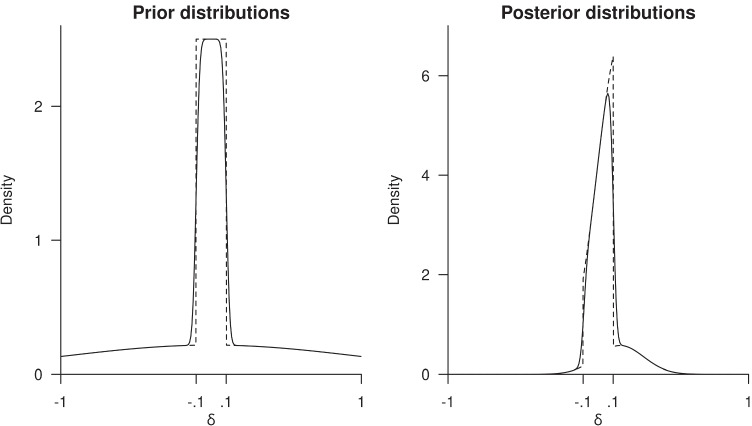
Approximating the hill-and-chimney prior by mollification. *Left panel*: The hill-and-chimney prior (*dashed line*) with *ρ*_0_ = .5 and *ε* = .1, and the mollified prior (*solid line*), for *α* = 600. *Right panel*: The hill-and-chimney posterior (*dashed line*) and the posterior based on the mollified prior (*solid line*). Based on data with *n* = 40, *δ* = .15, *σ* = 1, and *σ*_0_ = 1

As an example, consider a hill-and-chimney prior defined by *ρ*_0_ = .5 and *ε* = .1 (Fig. 7, left panel). As can be seen, the approximation looks very good, and has the advantage of being a smooth continuous function. Now we can again compute a number of specific probabilities under this posterior density function (Fig. 7, right panel). For the interval [−.1,.1], we now find a posterior odds of 3.81 compared to 5.59 for the hill-and-chimney prior. We also computed *P*(*δ* > 0|**y**) = .70, *P*(*δ* > 0.1|**y**) = .18, and *P*(*δ* > 0.3|**y**) = .04, while for the associated hill-and-chimney, these probabilities were .68, .14, and .04, respectively. Clearly, the odds differs quite a bit, but the probabilities are not that far off.

**Fig. 8:**
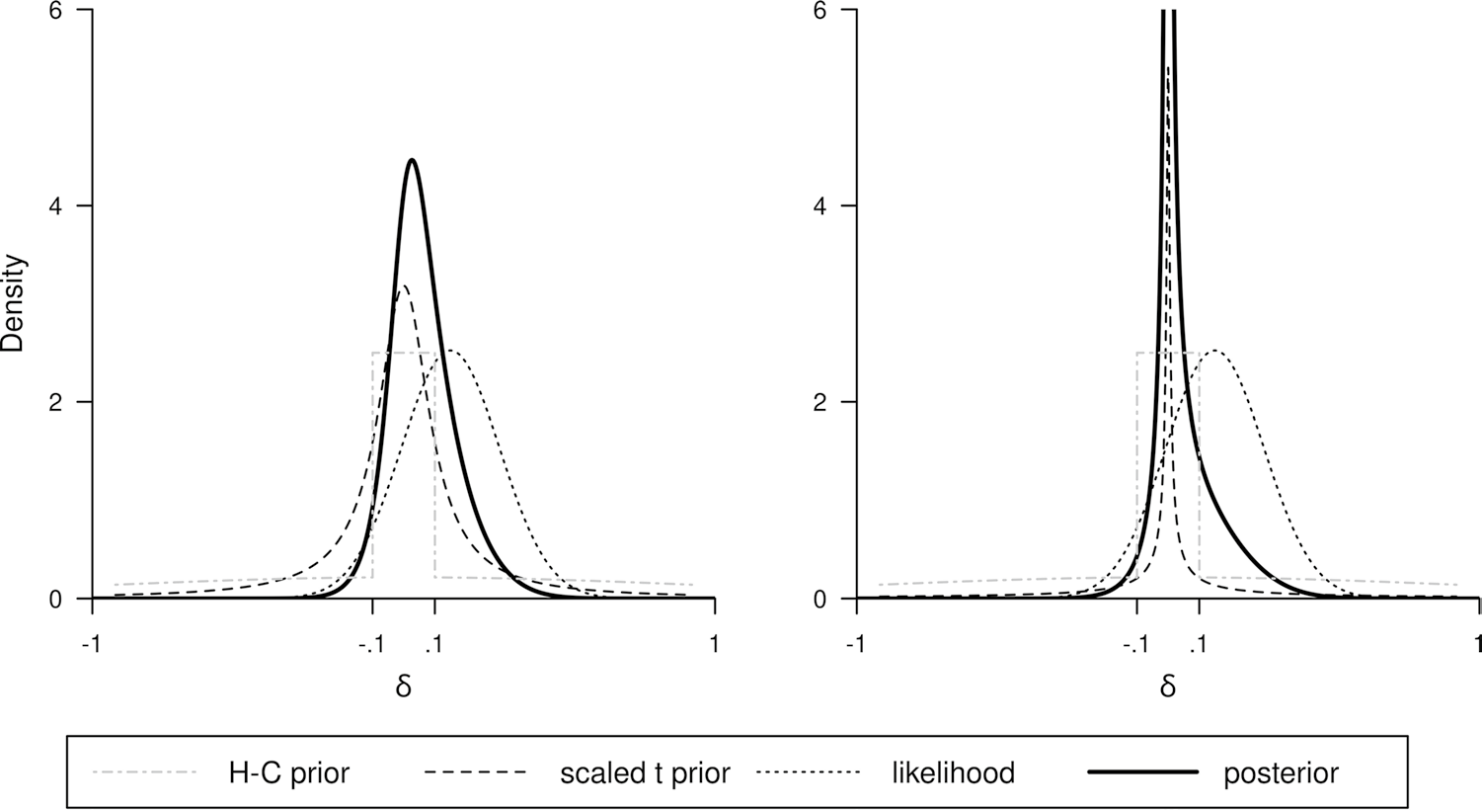
Results based on smooth peaked priors, based on data with *n* = 40, *δ* = .15, *σ* = 1, and *σ*_0_ = 1. The prior in the left panel is the *t*-distribution with *df* = 1, scaled by factor 10. The prior in the right panel is the *t*-distribution with *df* = .05, scaled by factor 50

When, in practice, a researcher wants to specify a prior with a strong peak around *δ* = 0, it may not be essential that it closely resembles a hill-and-chimney prior, as long as it gets the broad picture. Importantly, one should try to tune the choice of the hill-like and the chimney-like components in such a way that the variance ratio $r=\frac {Var_{\text {spike}}}{Var_{\text {slab}}}$ is much smaller than 1 (e.g., Frühwirth-Schnatter and Wagner, 2011). One alternative way of getting such priors is by using a scaled *t*-distribution (e.g., Wagner & Duller, 2012) with a low value for the degrees of freedom (e.g., see left panel in Fig. 8). For the present situation, after some trial-and-error, we found that the *t*-distribution with *df* = 0.05 and scale factor 50 (see right panel in Fig. 8) gave probabilities fairly close to those found for the (mollified) hill-and-chimney. The posterior odds was 4.06, while *P*(*δ* > 0|**y**) = .63 and *P*(*δ* > 0.1|**y**) = .18, which also are fairly close to those associated with the hill-and-chimney results (.68 and .14). In addition, and maybe more importantly, it should be realized that indeed the priors and posteriors for hill-and-chimney and for the smooth priors will lead to similar conclusions for all practical purposes, because by far most of the probability mass is concentrated between − .1 and .1.

### Using the spike-and-slab and hill-and-chimney priors

In the supplementary files available at https://osf.io/6rt9m/ we have included R scripts and models written in Stan (Stan Development Team, 2021) that allow using various priors, including the hill-and-chimney and approximations based on the normal and *t*-student distributions. We also include the necessary R code to apply the spike-and-slab model used in this paper.

## Decision-making

For the bridge between estimation and testing to be fully in place, we must also consider how decisions to be made depend on either inferential framework. First of all, we think that making discrete decisions is often not a necessity in social sciences. When a researcher is wondering about the merits of a hypothesis such as ${\mathscr{H}}_{0}:\delta =0$, we think that it is best to express uncertainty about the world through posterior distributions and posterior model probabilities and not necessarily to pick one out of the two hypotheses. The Bayes factor allows comparing the null hypothesis to one, competing, hypothesis, and that will lead to a factor, say, *BF*_01_ = 7.3. But there is little to learn from *one number only*. That the data are 7.3 times more probable under ${\mathscr{H}}_{0}$ than under one particular competing hypothesis is not very informative. On the other hand, concluding for instance that the posterior probability of ${\mathscr{H}}_{0}$ is .88 for equal prior odds, combined with a range of highly credible values, is much more telling. It is important to recognize that parameter estimation and its uncertainty and magnitude provide qualitatively different information from that provided by Bayes factors. Indeed, as we demonstrate, one can start right away with estimation and still have everything (parameter estimation, uncertainty, *and* testing information like the Bayes factor) in one go. Also here, estimation offers much more than the Bayes factor does, even if both can be unified as suggested in this paper.

Having said this, the need to make a binary decision is sometimes unavoidable (also, see Uygun Tunç et al., 2021). NHBT is often associated to decision heuristics aiding at choosing one of either hypotheses being compared, based on the strength of evidence brought about by the data (Jeffreys, 1961; Kass and Raftery, 1995). For example, it is common to use Bayes factor thresholds such as 3 or 10, so that for example if *BF*_01_ > 10 then the researcher is advised to retain the null hypothesis. By using estimation as outlined in this paper, this is of course doable. However, we do not find this procedure sensible in most circumstances. The main reason, as explained above, is that the Bayes factor (i.e., a measure of evidence from the data) differs from measures derived from posterior distributions (i.e., measures of updated belief). The latter reflect the updated probabilistic state of affairs concerning the hypotheses being compared and are arguably what researchers should focus on. The Bayes factor only coincides with the posterior odds if one prespecifies prior odds equal to 1. This assumption—that both hypotheses being compared are equally likely a priori—is often done in practice so Bayes factor users can somehow ‘get away with it.’ We do not think, however, that equal prior probabilities are always sensible. This is particularly true in the Bayesian framework which lends itself to incorporating prior knowledge in our inferences. As such, options for decision-making other than the Bayes factor should also be considered.

Luckily, using Bayesian estimation of parameters opens more possibilities for decision-making. As argued by Kruschke (2018), one can resort to the so-called HDI+ROPE criterion. This criterion is based on comparing the HDI for the parameter, which is the range of most credible parameter values, with the ROPE, which defines a range of parameter values that are considered equivalent to each other for all practical purposes. According to the HDI+ROPE criterion, the null hypothesis is accepted if the HDI falls entirely within the ROPE, the alternative hypothesis is accepted if the HDI falls entirely outside the ROPE, and a final decision is suspended otherwise. In this way, a hypothesis such as an interval null may be accepted ‘for practical purposes.’ Kruschke (2018, Supplement) considered decision-theoretic properties of the HDI+ROPE decision rule and showed that this rule is consistent and that it minimizes a certain prototypical loss function, while taking into account information that both the Bayes factor and the posterior odds fail to do.

A simple alternative is to use what Kruschke calls the ROPE alone procedure, suggested for instance by Wellek (2010, sections 2.4, 3.2). This approach consists of computing the posterior probability that the parameter is inside or outside the ROPE. A decision rule can then be set up by comparing these posterior probabilities to a predefined threshold, for instance, .95. Either hypothesis is to be retained if its associated posterior probability is over 95%, otherwise one remains undecided. Kruschke (2018) dismissed this procedure because it does not take into account the varying posterior density associated to the parameter values. However, one could also argue that the total probabilities within or outside the ROPE are all that should count for making a decision. Posterior probabilities are a valid measure of belief and thus we also think they can serve the purpose of decision-making.

Another option for decision-making is to focus on the posterior model odds. Unlike the Bayes factor, the posterior model odds are based on the posterior model probabilities, thus incorporate information from both the data and the prior model odds. The posterior model odds are readily available through the hill-and-chimney model (Liao et al., 2020), but some heuristic akin to the Bayes factor thresholds advocated for instance by Jeffreys (1961) would still need to be decided upon. Like the Bayes factor, the posterior odds also fail to consider useful information related to fully expressing model uncertainty that only a full access to the posterior distribution can disclose.

In cases where utilities can be attached to particular effect size values, one can compute the expected utility of, for instance, a treatment, which can then be offset against its costs. A very simple example is where *u*(*δ*) = 100,000 if *δ* > 0.2 and *u*(*δ*) = 0 otherwise, while the costs of the treatment are 10,000. For the right panel in Fig. 5, *P*(*δ* > 0.2|**y**) = .085, so the expected utility is 8,500. The costs hence exceed the expected benefit. If, however, *u*(*δ*) = 100,000 if *δ* > 0.2, *u*(*δ*) = 50,000 if *δ* ∈ [.1,.2], and *u*(*δ*) = 0 otherwise, then, using that *P*(*δ* ∈ [.1,.2]|**y**) = .099, the expected benefit is .099 × 50,000 + .085 × 100,000 = 13,450, which does exceed the costs. Computing the expected utility is beyond reach in NHBT, because NHBT only offers the Bayes factors.

## Discussion

We have seen that, with some effort, one can find a smooth prior that behaves reasonably similarly as a hill-and-chimney prior. In turn, the hill-and-chimney priors can approximate spike-and-slab priors arbitrarily closely as the chimney width tends to 0, but it seems difficult to justify such priors. This is because hill-and-chimney priors specify that the prior belief in *δ* equaling *ε* strongly differs from that of *δ* equaling *ε* + .000001, while for all practical purposes these two values are equal. Therefore, we think that, in order to justify a prior choice, the prior density should be a smooth continuous function. The mollified and other steep smooth priors discussed before are continuous and can be scaled such that they have a similar spiky shape as the hill-and-chimney prior, so they seem to be an excellent choice for researchers who actually endorse the spike-and-slab idea that there should be considerable probability mass on values very close to 0. Researchers could, for instance, find and tune spiky priors by adjusting the degrees of freedom and scale factor of the *t*-distribution at wish, and could find a prior sufficiently close to their ideal of a peaked mass close to 0. Having done so, they can compute a Bayes factor, but also any posterior probabilities they like, as well as HDIs, straightforwardly from the posterior distribution. In this way, one gets full insight in the posterior distribution, which will considerably enhance the understanding of the implications of the evidence provided by the Bayes factor (or any other single value) alone.

Following Rouder et al. (2018), a true unification between estimation and a variant of NHBT has been obtained. Actually, NHBT boils down to obtaining merely one particular probability statement based on aspects of the posterior distribution (i.e., the one that specifies the odds of being close to 0 against its opposite). However, having the posterior distribution, any so desired probability statement on the values of *δ* can be made, for instance, *P*(*δ* ∈ROPE|**y**), *P*(*δ* > 0|**y**), or *P*(*δ* > practically significant effect|**y**).

Maybe the most important feature of using smooth priors is that it allows one to move smoothly from strongly peaked priors to gradually flattening priors, and the researcher can choose the prior that is best justifiable according to his/her knowledge. Maybe in some or even many cases, it is unreasonable to hold a strong belief in near zero effect sizes. For instance, when testing treatments, often a long stage of development has preceded this, and it is to be expected that there actually is a substantial effect. In such cases, fairly flat priors, or even shifted priors around a nonzero effect size could be more reasonable. One might counter that this is ‘unfair’ as it leads to confirmation bias. However, the very idea of Bayesian analysis is that, in the data analysis, prior information can be introduced, which in the eyes of others indeed may be a bias with respect to those others’ norms. This prior is subjective, but it should be justified. In a way, one could say it is meant to ‘bias’/adjust a statement which one should make on the basis of the data alone, because on a particular phenomenon more information is available than just the present data. If there is reason to adjust a result towards small effects, then this can be done by putting a high mass on small values (as in the spike-and-slab prior), but if there is current knowledge about a substantial effect size, nothing is wrong with adjusting one’s results towards this. Typically, in such cases one will rarely find high posterior probabilities in close areas around 0. This, however, is then fully in agreement with current knowledge, and especially when one deals with small ROPEs, it will indeed be very unlikely that the population mean effect will be in there.

## Conclusions

The present paper elaborated on the unification of NHBT and Bayesian estimation described by Rouder et al. (2018). Following their approach, it did so for only one particular case of NHBT, that is, the one defining the alternative hypothesis in terms of $\delta \sim \mathcal {N}(0, {\sigma _{0}^{2}})$, while assuming *σ*, the standard deviation of the population scores *y*, known. The general reasoning was easy to illustrate for this situation, but it is rather unrealistic in practice, as *σ* will never be known, and can even be hard to guess. This is a limitation in our model. A more general approach is described by Rouder, Speckman, Sun, and Morey (2009) and uses, in addition to a different prior distribution for *δ* (viz. a Cauchy distribution), also a prior distribution for *σ*, which in computations, however is ‘integrated out.’ We do not know whether a particular class of hill-and-chimney priors exists that arbitrarily closely approximates this class of Cauchy priors, but we do believe that hill-and-chimney priors can be constructed that come close to it ‘for all practical purposes.’ Therefore, we believe that our reasoning is of general relevance. Likewise, we expect smooth spiky curves can be found to mimic the Cauchy prior used, while this can be combined with a prior for the unknown *σ*. Future research may thus aim at generalizing the hill-and-chimney paradigm to more designs. We think that the best way forward is to combine these ideas with MCMC sampling, as this will help broadening the implementation in practice.

Following Kruschke (2013), even an additional parameter for tuning the thickness of the tails of the prior could be used. We are convinced that such classes of priors can cater for diverse states of knowledge, that is, where there is strong knowledge that the effect size is close to 0 (or another value), as well as cases where only vague knowledge exists. It has been seen now that the spike-and-slab prior focuses only on the former case, which is fine, if justifiable. A more general approach, however, is offered by full posterior estimation, allowing for any type of prior, and compared to NHBT nothing is lost, but a lot gained.

## Appendix A: Useful lemma

The following lemma will be useful to derive the expression of the Bayes factor for the test used in the paper (Appendix B), the spike-and-slab posterior distribution (Appendix C), and the hill-and-chimney posterior distribution (Appendix D). It is a variation of a common result in Bayesian analysis based on conjugating a normal prior with normal data. In the remaining of the paper we will refer to it simply as ‘the Lemma.’

### **Lemma 1**

Let $y_{i} \sim \mathcal {N}(\sigma \delta , \sigma ^{2})$ for *i* = 1,…,*n* and *σ*^2^ known. If $\delta \sim \mathcal {N}(0, {\sigma _{0}^{2}})$ is the prior density for *δ*, then the (unnormalized) product of the prior for *δ* by the likelihood is

A1 $$\begin{array}{@{}rcl@{}} \text{prior}\times\text{likelihood} = \lambda \exp\left[-\frac{\left(\delta - \widetilde{\mu}\right)^{2}}{2\widetilde{\sigma}^{2}}\right], \end{array}$$

where $\lambda = \frac {1}{\sqrt {2\pi }\sigma _{0}} \left (\frac {1}{\sqrt {2\pi }\sigma }\right )^{n} \exp \left (-\frac {n\overline {y^{2}}}{2\sigma ^{2}}\right ) \exp \left [\frac {(n\overline {y}\sigma _{0})^{2}}{2\sigma ^{2}(1+n{\sigma _{0}^{2}})}\right ]$ does not depend on *δ* and the exponential is the kernel of a normal density with mean $\widetilde {\mu }=\frac {n\overline {y}{\sigma _{0}^{2}}}{\sigma (1+n{\sigma _{0}^{2}})}$ and variance $\widetilde {\sigma }^{2}=\frac {{\sigma _{0}^{2}}}{1+n{\sigma _{0}^{2}}}$.

### *Proof*

We have that

A2 $$\begin{array}{@{}rcl@{}} \text{prior}\times\text{likelihood} &=& \frac{1}{\sqrt{2\pi}\sigma_{0}}\exp\left(-\frac{\delta^{2}}{2{\sigma_{0}^{2}}}\right)\\ &&\times \left(\frac{1}{\sqrt{2\pi}\sigma}\right)^{n} \exp\left[-\frac{{\sum}_{i=1}^{n}(y_{i}-\sigma\delta)^{2}}{2\sigma^{2}}\right]. \end{array}$$

Next we combine the two exponentials in Eq. A2 and rewrite the argument of the resulting exponential as a quadratic function of *δ*:

A3 $$\begin{array}{@{}rcl@{}} \text{prior}\times\text{likelihood} &= &\frac{1}{\sqrt{2\pi}\sigma_{0}} \left(\frac{1}{\sqrt{2\pi}\sigma}\right)^{n} \exp\left(-\frac{\delta^{2}}{2{\sigma_{0}^{2}}} - \frac{{\sum}_{i=1}^{n}{y_{i}^{2}}}{2\sigma^{2}}\right.\\&&\left. - \frac{n\sigma^{2}\delta^{2}}{2\sigma^{2}} + \frac{2n\sigma\delta\overline{y}}{2\sigma^{2}} \right) \\ &=& \frac{1}{\sqrt{2\pi}\sigma_{0}} \left(\frac{1}{\sqrt{2\pi}\sigma}\right)^{n} \exp\left(-\frac{n\overline{y^{2}}}{2\sigma^{2}}\right) \\ & &\exp\left\{-\frac{1}{2}\left[\left(\frac{1+n{\sigma_{0}^{2}}}{{\sigma_{0}^{2}}}\right)\delta^{2} -\frac{2n\overline{y}}{\sigma}\delta\right]\right\}. \end{array}$$

Finally, we ‘complete the square’ of the quadratic function of *δ*:

$$\begin{array}{@{}rcl@{}} \text{prior}\times\text{likelihood} &=& \frac{1}{\sqrt{2\pi}\sigma_{0}} \left(\frac{1}{\sqrt{2\pi}\sigma}\right)^{n} \exp\left(-\frac{n\overline{y^{2}}}{2\sigma^{2}}\right) \exp\left[\frac{(n\overline{y}\sigma_{0})^{2}}{2\sigma^{2}(1+n{\sigma_{0}^{2}})}\right]\\ && \exp\left\{-\frac{1}{2}\left[\left(\frac{1+n{\sigma_{0}^{2}}}{{\sigma_{0}^{2}}}\right)\delta^{2} - \frac{2n\overline{y}}{\sigma}\delta + \left(\frac{n\overline{y}\sigma_{0}}{\sigma\sqrt{1+n{\sigma_{0}^{2}}}}\right)^{2}\right]\right\} \\ &=& \lambda \exp\left[-\frac{1}{2}\left(\frac{\sqrt{1+n{\sigma_{0}^{2}}}}{\sigma_{0}}\delta - \frac{n\overline{y}\sigma_{0}}{\sigma\sqrt{1+n{\sigma_{0}^{2}}}}\right)^{2}\right] \\ &=& \lambda \exp\left[-\frac{\left(\delta - \frac{n\overline{y}{\sigma_{0}^{2}}}{\sigma(1+n{\sigma_{0}^{2}})}\right)^{2}}{2\frac{{\sigma_{0}^{2}}}{1+n{\sigma_{0}^{2}}}}\right] \\ &=& \lambda\exp\left[-\frac{\left(\delta - \widetilde{\mu}\right)^{2}}{2\widetilde{\sigma}^{2}}\right]. \end{array}$$

This completes the proof. □

In particular, the Lemma implies that the posterior over the entire real numbers domain is the normal distribution $\mathcal {N}(\widetilde {\mu }, \widetilde {\sigma }^{2})$. The multiplicative constant *λ* is of value when Eq. A1 is constrained to a subset of the real numbers set, as it is the case in deriving the hill-and-chimney posterior distribution.

## Appendix B: Derivation of the Bayes factor

We want to derive the Bayes factor *B*_10_ to test ${\mathscr{H}}_{0}: \delta =0$ versus ${\mathscr{H}}_{1}: \delta \sim \mathcal {N}(0,{\sigma _{0}^{2}})$, for data **y** = {*y*_1_,…,*y*_*n*_} assumed normally distributed,

B1 $$y_{i}~\sim\mathcal{N}(\mu,\sigma^{2})=\mathcal{N}(\sigma\delta,\sigma^{2}), i=1,\ldots,n.$$

Observe that *δ* is the standardized effect size *δ* = *μ*/*σ* and that *σ*^2^ is assumed known. Here we will derive *B*_10_ directly by its definition.^8^ We have that

$$\begin{array}{@{}rcl@{}} B_{10} &= &\frac{p(\mathbf{y}|\mathcal{H}_{1})}{p(\mathbf{y}|\mathcal{H}_{0})} \\ &=& \frac{\displaystyle{\int}_{-\infty}^{\infty} p(\mathbf{y}, \delta|\mathcal{H}_{1})p(\delta|\mathcal{H}_{1})d\delta}{{\prod}_{i=1}^{n} \frac{1}{\sqrt{2\pi}\sigma}\exp\left[-\frac{(y_{i}-0)^{2}}{2\sigma^{2}}\right]}. \end{array}$$

Applying the Lemma to the integrand in the numerator we have that

B2 $$\begin{array}{@{}rcl@{}} B_{10} &=& \frac{\frac{1}{\sqrt{2\pi}\sigma_{0}} \left(\frac{1}{\sqrt{2\pi}\sigma}\right)^{n} \exp\left(-\frac{n\overline{y^{2}}}{2\sigma^{2}}\right) \exp\left[\frac{(n\overline{y}\sigma_{0})^{2}}{2\sigma^{2}(1+n{\sigma_{0}^{2}})}\right] \displaystyle{\int}_{-\infty}^{\infty} \exp\left[-\frac{\left(\delta - \widetilde{\mu}\right)^{2}}{2\widetilde{\sigma}^{2}}\right]d\delta}{\left(\frac{1}{\sqrt{2\pi}\sigma}\right)^{n}\exp\left(-\frac{n\overline{y^{2}}}{2\sigma^{2}}\right)} \\ &=& \frac{1}{\sqrt{2\pi}\sigma_{0}} \exp\left[\frac{(n\overline{y}\sigma_{0})^{2}}{2\sigma^{2}(1+n{\sigma_{0}^{2}})}\right] \sqrt{2\pi}\widetilde{\sigma} \\ &=& \frac{1}{\sqrt{1+n{\sigma^{2}_{0}}}} \exp\left[\frac{(n\overline{y}\sigma_{0})^{2}}{2\sigma^{2}(1+n{\sigma_{0}^{2}})}\right]. \end{array}$$

This equals the expression of *B*_10_ provided by Rouder et al. (2018, Footnote 2).

## Appendix C: Derivation of the spike-and-slab posterior distribution

Here we derive the posterior distribution for *δ*, given the prior defined in Eq. 1 and the same normal data model as given by Eq. B1. The proof consists of firstly computing the posterior probability of ${\mathscr{H}}_{0}$, and then assessing the posterior density for *δ*≠ 0. The posterior is:

C1 $$\left\{ \begin{array}{lll} p(\delta|\mathbf{y}) & = (1-\rho_{1})\times\frac{1}{\sqrt{2\pi}\sigma_{1}}\exp\left[-\frac{(\delta-\delta_{1})^{2}}{2{\sigma_{1}^{2}}}\right] & \text{, if } \delta\not=0 \\ P(\delta=0|\mathbf{y}) & = \rho_{1} & \text{, if } \delta=0 \end{array}, \right.$$

where

$$\begin{array}{@{}rcl@{}} \rho_{1} &= &\frac{\rho_{0}}{\rho_{0}+(1-\rho_{0})B_{10}} \\ \delta_{1} &=& \frac{n\overline{y}{\sigma_{1}^{2}}}{\sigma}\text{, and } \\ {\sigma_{1}^{2}} &=& \left(n+\frac{1}{{\sigma_{0}^{2}}}\right)^{-1}. \end{array}$$

Note that we did not use (Rouder et al., 2018)’s shortcut $d=\overline {y}/\sigma$. Hence this posterior for *δ* depends on $\overline {y}$, *n*, *σ*_0_, and *σ*.

We start by considering the basic equation that relates the Bayes factor with the prior and posterior odds (Eq. 3):

$$\frac{P(\mathcal{H}_{1}|\mathbf{y})}{P(\mathcal{H}_{0}|\mathbf{y})} = B_{10}\times \frac{P(\mathcal{H}_{1})}{P(\mathcal{H}_{0})}.$$

We know that $P({\mathscr{H}}_{0})=\rho _{0}$ and $P({\mathscr{H}}_{1})=1-\rho _{0}$ (see Eq. 1), and that $P({\mathscr{H}}_{1}|\mathbf {y}) = 1 - P({\mathscr{H}}_{0}|\mathbf {y})$. We therefore have the following:

$$\begin{array}{@{}rcl@{}} \frac{1-P(\mathcal{H}_{0}|\mathbf{y})}{P(\mathcal{H}_{0}|\mathbf{y})} &=& B_{10}\times \frac{1-\rho_{0}}{\rho_{0}} \\ \frac{1}{P(\mathcal{H}_{0}|\mathbf{y})} &=& 1 + B_{10}\times \frac{1-\rho_{0}}{\rho_{0}} \\ P(\mathcal{H}_{0}|\mathbf{y}) &=& \frac{\rho_{0}}{\rho_{0}+(1-\rho_{0})B_{10}} \\ P(\mathcal{H}_{0}|\mathbf{y}) &= &\rho_{1}. \end{array}$$

Thus we established that the posterior probability of ${\mathscr{H}}_{0}: \delta =0$ is equal to *ρ*_1_.

In order to find the posterior density *p*(*δ*|**y**) for *δ*≠ 0, we observe that this posterior is proportional to the product given by Eq. A1 from the Lemma, that is,

$$p(\delta|\mathbf{y}) \propto \exp\left[-\frac{\left(\delta - \widetilde{\mu}\right)^{2}}{2\widetilde{\sigma}^{2}}\right].$$

The total mass for *δ* ≠  0 should be (1 − *ρ*_1_), so we conclude that the seeked density must be

$$p(\delta|\mathbf{y}) = (1-\rho_{1})\frac{1}{\sqrt{2\pi}\widetilde{\sigma}}\exp\left[-\frac{\left(\delta - \widetilde{\mu}\right)^{2}}{2\widetilde{\sigma}^{2}}\right].$$

This is equal to the expression in Eq. C1 after noting that $\sigma _{1}=\widetilde {\sigma }$ and $\delta _{1}=\widetilde {\mu }$.

## Appendix D: The hill-and-chimney prior (Eq. 2)

In this section; we establish two results based on the hill-and-chimney prior (Eq. 2). Firstly, we derive the expression of constant *k* in Eq. 2. Next, we derive the expression of the posterior distribution for *δ*, given the hill-and-chimney prior and the normal data model from Eq. B1.

### Constant *k*

In order to find *k*, it is useful to consider the following relation:

D1 $$\frac{1-\rho_{0}}{2} = {\int}_{-\infty}^{-\varepsilon} p(\delta) d\delta = k{\Phi}\left(-\frac{\varepsilon}{\sigma_{0}}\right).$$

This first equality holds because the hill-and-chimney prior is symmetric around 0 and the chimney area is by definition equal to *ρ*_0_ (since ${\int \limits }_{-\varepsilon }^{\varepsilon }\frac {\rho _{0}}{2\varepsilon } d\delta = \rho _{0}$). Therefore, the area under the slab (= 1 − *ρ*_0_) is evenly divided across the intervals $(-\infty ,-\varepsilon )$ and $(\varepsilon , \infty )$. The second equality holds because the slab is based on the (rescaled) normal density $\mathcal {N}(0, {\sigma _{0}^{2}})$. Solving Eq. D1 with respect to *k* implies that

D2 $$k=\frac{1-\rho_{0}}{2{\Phi}\left(-\frac{\varepsilon}{\sigma_{0}}\right)}.$$

### Derivation of the hill-and-chimney posterior distribution

We first find the unnormalized posterior (i.e., the product of the prior by the likelihood), and then we find the normalization factor, which is the marginal likelihood. Jointly these will lead to the posterior distribution.

#### The unnormalized posterior

We must consider two situations, namely, *δ* ∈ [−*ε*,*ε*] and *δ*∉[−*ε*,*ε*].

When *δ* ∈ [−*ε*,*ε*] we have that

D3 $$\begin{array}{@{}rcl@{}} \text{prior}\times\text{likelihood} &=& p(\delta)L(\delta|\mathbf{y})\\ &=& \frac{\rho_{0}}{2\varepsilon}\left(\frac{1}{\sqrt{2\pi}\sigma}\right)^{n} \exp\left[-\frac{{\sum}_{i=1}^{n}(y_{i}-\sigma\delta)^{2}}{2\sigma^{2}}\right]. \end{array}$$

The exponential in Eq. D3 can be decomposed as follows:

$$\begin{array}{@{}rcl@{}} \exp\left[-\frac{{\sum}_{i=1}^{n}(y_{i}-\sigma\delta)^{2}}{2\sigma^{2}}\right] &=& \exp\left[-\frac{1}{2\sigma^{2}} \left(n\sigma^{2}\delta^{2} - 2\sum\limits_{i=1}^{n}y_{i}\sigma\delta + \sum\limits_{i=1}^{n}{y_{i}^{2}} \right) \right] \\ &=& \exp\left[-\frac{n}{2\sigma^{2}} \underbrace{\left(\sigma^{2}\delta^{2} - 2\overline{y}\sigma\delta + \overline{y^{2}} \right)} \right]. \end{array}$$

Next we complete the square of the quadratic function in *δ*:

D4 $$\begin{array}{@{}rcl@{}} \exp\left[-\frac{n}{2\sigma^{2}} \left(\sigma^{2}\delta^{2} - 2\overline{y}\sigma\delta + \overline{y^{2}} \right) \right] &=& \exp\left[-\frac{n}{2\sigma^{2}} \left(\sigma^{2}\delta^{2} - 2\overline{y}\sigma\delta + \overline{y}^{2} - \overline{y}^{2} + \overline{y^{2}} \right) \right] \\ &=& \beta_{1}\exp\left[-\frac{n}{2\sigma^{2}} \left(\sigma\delta - \overline{y} \right)^{2} \right] \\ &=& \beta_{1}\exp\left[-\frac{n}{2} \left(\delta - \frac{\overline{y}}{\sigma} \right)^{2} \right], \end{array}$$

where $\beta _{1} = \exp \left [-\frac {n}{2\sigma ^{2}}\left (\overline {y^{2}} - \overline {y}^{2}\right )\right ]$. Replacing the exponential in Eqs. D3 by D4 finally leads to

D5 $$\begin{array}{@{}rcl@{}} \text{prior}\times\text{likelihood} &= \frac{\rho_{0}}{2\varepsilon}\left(\frac{1}{\sqrt{2\pi}\sigma}\right)^{n} \beta_{1}\exp\left[-\frac{n}{2} \left(\delta - \frac{\overline{y}}{\sigma} \right)^{2} \right]. \end{array}$$

The rightmost exponential factor in Eq. D5 is the kernel of a normal density with mean $\mu _{\text {chimney}}=\frac {\overline {y}}{\sigma }$ and variance $\sigma _{\text {chimney}}^{2}=\frac {1}{n}$.

When *δ* ∉ [−*ε*, *ε*] we can use the Lemma:

D6 $$\begin{array}{@{}rcl@{}} \text{prior}\times\text{likelihood} &=& p(\delta)L(\delta|\mathbf{y})\\ &=& k\lambda\exp\left[-\frac{(\delta - \widetilde{\mu})^{2}}{2\widetilde{\sigma}^{2}}\right]. \end{array}$$

We conclude that the unnormalized posterior is given by Eq. D5 when *δ* = 0 and by Eq. D6 when *δ* ≠  0.

#### The marginal likelihood

The marginal likelihood is given by ${\int \limits }_{-\infty }^{\infty }\text {prior}\times \text {likelihood}\, d\delta$, by definition. This integral can be computed as a sum of two terms, namely,

D7 $$\begin{array}{@{}rcl@{}} {\int}_{\delta\in[-\varepsilon,\varepsilon]}\text{prior}\times\text{likelihood}\, d\delta + {\int}_{\delta\not\in[-\varepsilon,\varepsilon]}\text{prior}\times\text{likelihood}\, d\delta. \end{array}$$

The first term of Eq. D7 is equal to (recall Eq. D5)

D8 $$\begin{array}{@{}rcl@{}} {\int}_{\delta\in[-\varepsilon,\varepsilon]}\text{prior}\times\text{likelihood}\, d\delta &=& \frac{\rho_{0}}{2\varepsilon}\left(\frac{1}{\sqrt{2\pi}\sigma}\right)^{n} \beta_{1} \sqrt{2\pi}\sigma_{\text{chimney}}\\ & &\left[ {\Phi}\left(\frac{\varepsilon - \mu_{\text{chimney}}}{\sigma_{\text{chimney}}}\right) - {\Phi}\left(\frac{-\varepsilon - \mu_{\text{chimney}}}{\sigma_{\text{chimney}}}\right) \right]. \end{array}$$

The second term of Eq. D7 is equal to (recall Eq. D6)

D9 $$\begin{array}{@{}rcl@{}} {\int}_{\delta\not\in[-\varepsilon,\varepsilon]}\text{prior}\times\text{likelihood}\, d\delta &= k\lambda\sqrt{2\pi}\widetilde{\sigma} \left[ 1 - {\Phi}\left(\frac{\varepsilon - \widetilde{\mu}}{\widetilde{\sigma}}\right) + {\Phi}\left(\frac{-\varepsilon - \widetilde{\mu}}{\widetilde{\sigma}}\right) \right]. \end{array}$$

#### The hill-and-chimney posterior distribution

Finally, the hill-and-chimney posterior distribution is equal to

D10 $$\begin{array}{@{}rcl@{}} \frac{\text{prior}\;\times\;\text{likelihood}}{{\int}_{-\infty}^{\infty}\;\text{prior}\;\times\;\text{likelihood}\; d\delta}. \end{array}$$

The numerator of Eq. D10 is equal to either Eq. D5 (for *δ* ∈ [−*ε*, *ε*]) or Eq. D6 (for *δ* ∉ [−*ε*, *ε*]). The denominator of Eq. D10 is given by Eq. D7.

### The hill-and-chimney prior converges to the spike-and-slab

We first show that the hill part of the hill-and-chimney prior density converges to the slab part of the spike-and-slab prior density as *ε* converges to 0. Observe that both densities are rescaled versions of the normal density $\mathcal {N}(0, {\sigma _{0}^{2}})$. What distinguishes both expressions is the rescaling factor, which equals *k* (Eq. D2) for the hill-and-chimney prior and (1 − *ρ*_0_) for the spike-and-slab prior. Thus, all we need is to show is that $\lim _{\varepsilon \rightarrow 0}k = 1-\rho _{0}$. And indeed,

$$\underset{\varepsilon\rightarrow 0}{\lim} k = \underset{\varepsilon\rightarrow 0}{\lim} \frac{1-\rho_{0}}{2{\Phi}\left(-\frac{\varepsilon}{\sigma_{0}}\right)} = \frac{1-\rho_{0}}{2{\Phi}(0)} = 1-\rho_{0}.$$

Moreover, since the probability for the interval [−*ε*,*ε*] (i.e., ${\int \limits }_{-\varepsilon }^{\varepsilon }\frac {\rho _{0}}{2 \varepsilon } = \rho _{0}$) is constant for any value of *ε*, we conclude that the probability for the interval [−*ε*,*ε*] converges to the probability mass *ρ*_0_ at *δ* = 0 as $\varepsilon \rightarrow 0$. That is, in the limit, *P*(*δ* = 0) = *ρ*_0_, which is the expression for the spike (Eq. 1). This concludes the argument.

## Footnotes

1 The slab of the spike-and-slab prior used by Mitchell and Beauchamp (1988) is the uniform distribution on a finite symmetric interval around 0. It is therefore different from Eq. 1 but the underlying principle is the same.

2 In this paper, we use the notation *P*() for probabilities and *p*() for probability densities.

3 Even here one can debate this, if one takes the position that the population parameters should refer to measurements as they actually are being taken (rather than idealized constructs): The procedure of computing the effect size will then not be flawless, and even when a complete full population would be studied, due to operationalization issues, it will be close to impossible that the mean effect size is exactly 0, not even if purported precognition really has no effect whatsoever.

4 In fact, we used R code from Richard Morey that produces exactly the same results as JASP. Code available at https://osf.io/6rt9m/.

5 As noted by a reviewer, not all Bayes factors allow for prior odds adjustment. One example is based on Bayes factors for inequality constrained hypotheses, which require to use 50/50 prior odds (Klugkist, Laudy, & Hoijtink, 2005).

6 As noted by a reviewer, the decision on which prior to use should be ultimately based more on theoretical reasons and information available at the time and less on tradition or convenience.

7 Our function was derived from the *standard mollifier* function (Evans 2010, Section C.5) given by $f(\delta )=k\exp \left (\frac {1}{\delta ^{2}-1}\right )$ in the interval (− 1, 1) and 0 elsewhere. First we scaled the function to the interval $(-\varepsilon_{\sigma_{0}}, \varepsilon_{\sigma_{0}})$: $f_{2}(\delta )=k\exp \left [\frac {1}{(\delta /\varepsilon _{\sigma _{0}})^{2}-1}\right ]$. Multiplying the numerator and denominator of the argument of the exponential by $\left (\frac {\varepsilon _{\sigma _{0}}}{\alpha }\right )^{2}$ leads to the following equivalent function in $(-\varepsilon _{\sigma _{0}}, \varepsilon _{\sigma _{0}})$: $f_{3}(\delta )=k\exp \left [\frac {(\varepsilon _{\sigma _{0}}/\alpha )^{2}}{(\delta /\alpha )^{2}-(\varepsilon _{\sigma _{0}}/\alpha )^{2}}\right ]$. Equation 6 results from *f*_3_(*δ*) after dropping the numerator of the argument (which is independent from *δ*) and adding a numerical *adjustment* given by $\left (\frac {\alpha }{\varepsilon _{\sigma _{0}}}\right )^{2}$. The adjustment counters the quick convergence of the term $\frac {1}{(\delta /\alpha )^{2}-(\varepsilon _{\sigma _{0}}/\alpha )^{2}}$ to $-\infty$ as *α* increases. Observe that the adjustment is independent from *δ* so it does not affect the mollifier once the normalizing constant *k* is computed.
